# Outpatient parenteral antibiotic therapy with daptomycin: insights from a patient registry

**DOI:** 10.1111/j.1742-1241.2008.01824.x

**Published:** 2008-08

**Authors:** W J Martone, K C Lindfield, D E Katz

**Affiliations:** Cubist Pharmaceuticals, Inc.Lexington, MA, USA

## Abstract

**Aim:**

To compare and contrast the characteristics and clinical outcomes of patients who have received daptomycin as outpatients and inpatients.

**Methods:**

The Cubicin Outcomes Registry and Experience (CORE) is a retrospective chart review of patients who have received daptomycin in participating institutions. Patients treated in 2005 were included in this analysis. Demographic characteristics and clinical outcomes (success = cured + improved) were compared among patients who received outpatient parenteral antibiotic therapy (OPAT) and patients who had received inpatient parenteral antibiotic therapy (IPAT).

**Results:**

Of 1172 patients reported by 52 CORE 2005 participating institutions/investigators, 949 (81.0%) patients were evaluable: 539 (56.8%) received OPAT (OPAT patients), and 410 (43.2%) received only IPAT (IPAT patients). Of the 539 OPAT patients, 273 (50.6%) also received some IPAT, usually preceding OPAT therapy. Successful outcomes [no. of successes/(no. of successes + no. of failures)] for OPAT patients vs. IPAT patients were 94.6% and 86.3% respectively (chi-square test, p < 0.001). OPAT patients were younger, had fewer underlying diseases, were clinically stable, and had fewer adverse events than IPAT patients.

**Conclusions:**

Outpatient parenteral antibiotic therapy use was common (539/949 or 56.8%) among patients in CORE 2005. Clinical outcomes among OPAT patients appeared at least as good as or better than IPAT patients. Better outcomes among OPAT patients were most likely because of patient selection for OPAT. Additional studies should focus on clinical characteristics of patients who would be ideal candidates for daptomycin OPAT.

**What's known**

Outpatient parenteral antibiotic therapy (OPAT) is becoming increasingly important for treating patients requiring intravenous antibiotic therapy. Daptomycin, a novel lipopeptide intravenous antibiotic with rapid bactericidal activity against gram-positive pathogens, has many features, including once-daily dosing, that make it attractive for OPAT therapy. Little information is available about the outcomes of patients who have received daptomycin OPAT.

**What's new**

Among a cohort of patients in the 2005 Cubicin Outcomes Registry and Experience (CORE) who received daptomycin for gram-positive infections, outcomes among patients receiving OPAT were as good as or better than those receiving only inpatient parenteral antibiotic therapy (IPAT). Patient selection for OPAT was probably a major explanation of this finding. The frequency of antibiotic discontinuations for adverse events during OPAT therapy compared favourably to literature reports of other antibiotics used in OPAT.

## Introduction

Since its introduction in the 1980s, the frequency of outpatient parenteral antibiotic therapy (OPAT) in the USA has been increasing (1,2). Advantages of OPAT include reduced hospital stays and patient convenience. Guidelines for OPAT incorporate criteria for proper patient and antimicrobial selection (3,4). Antimicrobials with long half-lives are extensively prescribed; those that can be administered once a day reduce disruption of the patients’ daily activities and limit potential complications (5).

Daptomycin (Cubicin®; Lexington, MA, USA), a novel lipopeptide antibiotic with rapid *in vitro* bactericidal activity against gram-positive pathogens, has been approved for treatment of the following conditions: (i) complicated skin and skin structure infections (cSSSI) because of susceptible strains of certain gram-positive microorganisms including methicillin-resistant *Staphylococcus aureus* (MRSA), at a dose of 4 mg/kg per day intravenously and (ii) bacteremia and right-sided endocarditis because of MRSA and methicillin-sensitive *S. aureus*, at a dose of 6 mg/kg per day intravenously (6,7). Clinical studies have demonstrated the efficacy of daptomycin in these indications, but little is known about the postapproval experience with this drug administered as OPAT. Data from patients treated in 2005 in the Cubicin Outcomes Registry and Experience (CORE 2005), a retrospective observational chart review of patients who had received daptomycin, were used in this analysis. Characteristics and clinical outcomes of patients enrolled in CORE 2005 who received OPAT or inpatient parenteral antibiotic therapy (IPAT) were compared and contrasted.

## Methods

### General description of CORE and retrospective chart review

A standardised case report form and protocol were used to collect demographic and clinical information on patients who had been treated with daptomycin in 52 separate institutions in the USA during the time period of January–December 2005. A mix of acute care inpatient facilities, outpatient infusion centres, and home infusion centres were selected based on daptomycin use and willingness to participate in the registry. After institutional review board approval, demographic and clinical information was collected from patient medical records by trained study investigators in each site. Demographic information included patient gender, age group, weight, setting in which the patient was located in the 48 h prior to initiating daptomycin therapy, and patient location at the time of daptomycin administration (i.e. inpatient or outpatient setting). Clinical information included history of underlying diseases, infection types for which daptomycin was prescribed, dose and frequency of daptomycin administration, prior antibiotic therapy, clinical response to treatment and adverse events. A maximum of 30 records were reviewed from each institution. In cases where institutions had more than 30 patients treated with daptomycin, investigators were instructed to randomly select 30 patient records for review. Eligible patients received at least one dose of daptomycin and were not part of a controlled clinical trial.

### Clinical outcomes

Clinical outcomes were defined as:

- Cure: Clinical signs and symptoms resolved and/or no additional antibiotic therapy was necessary, or the infection cleared with a negative culture reported at the end of daptomycin therapy.
- Improved: Partial resolution of clinical signs and symptoms and/or additional antibiotic therapy was necessary at the end of daptomycin therapy.
- Failure: Inadequate response to daptomycin therapy; or, resistant, worsening or new/recurrent signs and symptoms; or, there was a need for a change in antibiotic therapy; or, a positive culture was reported at the end of therapy.
- Non-evaluable: Unable to determine response at the end of daptomycin therapy because the record(s) did not contain adequate information.

Non-evaluable patients were excluded from the analysis. Infections in patients having more than one primary infection for which daptomycin was prescribed were arranged in a hierarchical order according to clinical severity, specifically, endocarditis > bacteremia > osteomyelitis > other invasive infections > cSSSI > uncomplicated skin and skin structure infections (ucSSSI).

### Statistical analysis

For purposes of analysis, patients classified as ‘cure’ or ‘improved’ were combined (i.e. success). The per cent success was calculated as follows: no. of successes/(no. of successes + no. of failures). Categorical data were analysed using Fisher's exact test or chi-square test as appropriate. If overall categorical statistical tests were significant (p < 0.05) then individual (subset) comparisons were performed. No adjustments were made for multiple comparisons. Continuous variables were tested by the *t*-test or median test as appropriate.

## Results

### Overview

Of 1172 patients reported by 52 CORE 2005 participating institutions/investigators, 949 (81.0%) patients were evaluable. OPAT institutions/investigators (e.g. infusion centres) accounted for 19 (36.5%) of the 52 participating institutions/investigators, and for 539 (56.8%) of the 949 patients in this analysis.

The 223 unevaluable patients were comprised of 210 patients initially classified by institutional investigators as unevaluable or ‘other’, and an additional 13 patients (two cured, 10 improved and one failure) in whom either OPAT and/or IPAT daptomycin dosing was missing.

### Characteristics of evaluable and unevaluable patients (data not shown)

A comparison of evaluable and unevaluable patients revealed that a significantly higher proportion of unevaluable patients received IPAT, had bacteremia, and had more underlying diseases. Conversely, a higher proportion of evaluable patients had no underlying diseases, had cSSSI or had ucSSSI.

### OPAT and IPAT patient groupings

Among the evaluable patients, 266 (28.0%) received OPAT only, 410 (43.2%) received IPAT only, and 273 (28.8%) received IPAT plus OPAT. The majority of patients in the latter category included patients who continued their daptomycin intravenous therapy as outpatients. Overall, outcome (i.e. per cent success) for the patients who received only OPAT (93.6%) when compared with patients who received IPAT plus OPAT (95.6%) was not statistically significantly different (p = 0.34, Fisher's exact test, two tailed). Therefore, unless indicated otherwise, for the purposes of further analysis patients who received any OPAT were combined and are referred to as OPAT patients (*n* = 539). Patients who received only IPAT are referred to as IPAT patients (*n* = 410).

### Demographics and other characteristics of evaluable patients

In contrast to IPAT patients, OPAT patients tended to be female, younger and community residents in the 48 h prior to initiation of daptomycin therapy; to have only one primary infection for which daptomycin was prescribed; and to have fewer underlying diseases (Table 1). The inpatient healthcare setting in which daptomycin was first administered differed among OPAT and IPAT patients: 31 (5.8%) of 539 OPAT patients versus 93 (22.7%) of 410 IPAT patients were located in medical or surgical intensive care or coronary care units at the time daptomycin was first administered (p < 0.0001, chi-square test).

**Table 1:** Demographics and characteristics by location of daptomycin therapy

Characteristic	OPAT patients	IPAT patients	Total	p-value*
Female gender	236/538 (43.9)†	226/409 (55.3)	462/947 (48.8)	<0.001
Age > 50	305/539 (56.6)	259/410 (63.2)	564/949 (59.4)	0.041
Prior antibiotics‡	411/535 (76.8)	319/408 (78.2)	730/943 (77.4)	0.620
Consulted infectious diseases	483/539 (89.6)	378/410 (92.2)	861/949 (90.7)	0.174
Community location§	275/536 (51.3)	154/410 (37.6)	429/946 (45.4)	<0.001
Any healthcare location§	251/536 (46.8)	254/410 (62.0)	505/946 (53.4)	<0.001
No. of primary infections > 1¶	61/539 (11.3)	67/410 (16.3)	128/949 (13.5)	0.025
**No. of underlying diseases**				
Mean ± (SD)	1.89 (1.49)	2.72 (1.45)	2.25 (1.47)	<0.001**
Median (minimum, maximum)	2 (0,5)	3 (0,5)	2 (0,5)	<0.001††

* Chi-square test unless otherwise indicated. †No. with characteristic/no. of OPAT patients or IPAT patients (%). Denominators are not all equal because of missing values. ‡Potentially effective against gram-positive pathogens. §In the 48 h period before first daptomycin use. ¶For which daptomycin was prescribed; up to two primary infections were captured.

** Student *t*-test. ††Median test. OPAT, outpatient parenteral antibiotic therapy; IPAT, inpatient parenteral antibiotic therapy.

### Type of infection, location, pathogens and clinical outcomes of daptomycin treatment

Proportionately more IPAT patients had bacteremia, and more OPAT patients had osteomyelitis and ucSSSI (Table 2). Staphylococci accounted for 595 (76.1%) of 782 infections for which a pathogen was identified; *S. aureus* accounted for over half of the staphylococcal infections. Enterococci were the second most common group of pathogens, accounting for 124 (15.9%) of the 782 infections overall.

**Table 2:** Infection type by location of daptomycin therapy

Type of infection	OPAT patients, no. (% of OPAT patients)	IPAT patients, no. (% of IPAT patients)	Total no. (% of total infections)	p-value*
Endocarditis	14 (2.6%)	15 (3.6%)	29 (3.1%)	0.349†
Bacteremia	73 (13.5%)	143 (34.8%)	216 (22.8%)	< 0.001
Osteomyelitis	98 (18.2%)	18 (4.4%)	116 (12.2%)	< 0.001†
Other	78 (14.5%)	75 (18.3%)	153 (16.1%)	0.113
cSSSI	177 (32.8%)	123 (30.0%)	300 (31.6%)	0.351
ucSSSI	99 (18.4%)	36 (8.8%)	135 (14.2%)	< 0.001
Total	539 (56.8%)‡	410 (43.2%)‡	949 (100%)	< 0.001

* Differences in proportion of infection type for OPAT patients vs. IPAT patients. Chi-square test unless otherwise indicated. Overall table chi-square p < 0.001. †Fisher's exact test. ‡no. (% of total infections). OPAT, outpatient parenteral antibiotic therapy; IPAT, inpatient parenteral antibiotic therapy; cSSSI, complicated skin and skin structure infections; ucSSSI, uncomplicated skin and skin structure infections.

Overall, success rates were higher in OPAT patients (510/539 or 94.6%) than in IPAT patients (354/410 or 86.3%) (p < 0.001, chi-square test) (Table 3). OPAT patients with bacteremia and endocarditis had significantly higher success rates, and those with cSSSI had marginally higher success rates, than IPAT patients with similar infections (Table 3).

**Table 3:** Clinical outcome (success rates) by infection type and location of daptomycin therapy*

Infection type	OPAT patients	IPAT patients	Total	p-value*
Endocarditis	13/14 (92.9%)†	6/15 (40.0%)	19/29 (65.5%)	0.005
Bacteremia	71/73 (97.3%)	116/143 (81.2%)	187/216 (86.6%)	< 0.001
Osteomyelitis	89/98 (90.8%)	18/18 (100%)	107/116 (92.2%)	0.351
Other	71/78 (91.0%)	70/75 (93.3%)	141/153 (92.2%)	0.766
cSSSI	169/177 (95.5%)	110/123 (89.43%)	279/300 (93.0%)	0.064
ucSSSI	97/99 (98.0%)	34/36 (94.4%)	131/135 (97.0%)	0.289
All	510/539 (94.6%)	354/410 (86.3%)	864/949 (91.0%)	< 0.001‡

* Differences in proportion of success for OPAT patients vs. IPAT patients. Fisher's exact test (two tailed) unless otherwise indicated. Overall table chi-square p < 0.0001. †No. of successes/(no. of successes + no. of failures) (% success). ‡Chi-square test. OPAT, outpatient parenteral antibiotic therapy; IPAT, inpatient parenteral antibiotic therapy; cSSSI, complicated skin and skin structure infections; ucSSSI, uncomplicated skin and skin structure infections.

### Duration of daptomycin therapy

Patients who received only OPAT received a median of 17 days (range: 3–144) and IPAT patients received a median of 7 days (range: 1–153) of daptomycin therapy. Patients who received IPAT plus OPAT (i.e. 273 of the OPAT patients) received a median of 5 days (range: 1–56) and 20 days (range: 3–358) of IPAT and OPAT daptomycin therapy respectively.

### Adverse events

A total of 216 adverse events were reported in 131 (13.8%) of the 949 patients. Fifty (9.3%) of 539 OPAT patients and 81 (19.8%) of 410 IPAT patients experienced at least one adverse event (chi-square test, p < 0.0001). Sixty-five patients experienced 89 adverse events evaluated as possibly related to daptomycin therapy: 31 (5.8%) of 539 OPAT patients and 34 (8.3%) of 410 IPAT patients (chi-square test, p = 0.12). A significantly higher proportion of IPAT patients (10 of 410 or 2.4%) vs. OPAT patients (one of 539 or 0.2%) experienced possibly related diarrhoea (p = 0.001, Fisher's exact test, two tailed). Conversely, 14 of 539 (2.6%) OPAT patients and four of 410 (1.0%) IPAT patients experienced creatine phosphokinase elevations or myalgias (p = 0.08, Fisher's exact test, two tailed).

Daptomycin therapy was stopped because of one or more adverse events in 19 (3.5%) OPAT patients and 23 (5.6%) IPAT patients (p = 0.122, chi-square test).

## Discussion

Outpatient parenteral antibiotic therapy has become established as a safe and effective alternative to IPAT for selected patients in the USA. Several brief historical reviews have highlighted the evolution of OPAT over the past few decades, culminating in current estimates of over 250,000 OPAT patients annually (1,2). A wide variety of medical conditions have been treated with OPAT and recommendations for patient selection and management have been published (3,5,8–11).

A number of reviews have focused on the characteristics of antimicrobials which would enable optimal outpatient management. These characteristics include appropriate pharmacokinetic and pharmacodynamic profiles such as long half-life allowing for infrequent dosing, chemical stability, short infusion time, safety, and for certain infections, bactericidal activity (4,12). In this respect, several characteristics of daptomycin appear favourable to its use as OPAT: a half-life of 8–9 h allowing for once-daily dosing, concentration-dependent bactericidal activity, prolonged postantibiotic effect (> 6 h), relative stability after reconstitution, short infusion time of 30 min, and low frequency of injection site reactions (13–16).

In this analysis, OPAT patients had significantly higher success rates than IPAT patients. Undoubtedly, a major explanation for this finding was patient selection; compared with IPAT patients, OPAT patients were younger and were less seriously ill. In addition, OPAT patients had fewer adverse events than IPAT patients. In comparison to reports in the literature, the percentage of OPAT patients in whom daptomycin was stopped early because of an adverse event, 3.5%, is similar, if not lower than the percentage of OPAT patients in whom courses of other antibiotics were stopped because of adverse events (range: 2.9–9.8%) (5).

This analysis had a number of limitations including its retrospective nature, moderate number of unevaluable patients, non-representative sampling of inpatient and outpatient centres participating in CORE 2005, lack of information on comparator antibiotic treatments, and lack of randomisation. Significant outcome differences in favour of OPAT may have been magnified if unevaluable patients were included in the analysis. Unevaluable patients were disproportionately IPAT patients, had bacteremia, and had more (serious) underlying diseases, factors which might reasonably be associated with worse outcomes. Finally, the ideal study comparing OPAT and IPAT outcomes should be randomised (17). That is, patients who would otherwise qualify for OPAT therapy would be randomised into OPAT and IPAT groups.

In conclusion, OPAT use was common (539/949 or 56.8%) among patients in CORE 2005. Clinical outcomes among OPAT patients appeared at least as good as or better than IPAT patients. Better outcomes among OPAT patients were most likely because of patient selection for OPAT. Additional studies should focus on clinical characteristics of patients who would be ideal candidates for daptomycin OPAT.
